# Has China’s dairy market recovered from the 2008 melamine scandal? Quantitative evidence and policy implications

**DOI:** 10.3168/jdsc.2026-1075

**Published:** 2026-08-06

**Authors:** Qingbin Wang, Jana Murphy, Yang Zou

**Affiliations:** 1 Department of Community Development and Applied Economics, University of Vermont, Burlington, VT 05405; 2 School of Economics, Nankai University, Tianjin 300071, China

## Abstract

This study assesses the 2008 melamine scandal's effects on China's dairy market and derives policy implications. Specifically, time-series data from 1995 to 2024 are used to assess the changes and trends in China's milk production, dairy product imports, total dairy product consumption, and self-sufficiency ratio through graphical analysis and then to assess the melamine scandal's effects on China's milk production and dairy product consumption via regression analysis. This study suggests 3 major findings. First, although China's total milk production increased steadily by an average of 2.31 million metric tonnes (mmt) per year from 1995 to 2008, it fluctuated around a flat line from 2009 to 2018, and the major factor for the 10-year stagnation was very likely the melamine scandal. Second, the melamine scandal's effects on China's total dairy product consumption were relatively limited and the significant gaps between domestic consumption and production were met through increased dairy product imports, from 3.15 mmt in 2008 to 16.47 mmt in 2018, and reached a record high of 22.65 mmt in 2021. Third, the rapid increase in China's imports reduced its dairy product self-sufficiency ratio steadily from 91.12% in 2008 to 62.52% in 2021, resulting in significant policy concerns about whether China depended too much on dairy product imports. Although the melamine scandal severely damaged consumer confidence in domestic dairy products and it took 10 yr for China's milk production to recover to the 2008 level, China must take serious measures to improve its food safety and avoid safety scandals and outbreaks. Also, China is likely to remain as a large dairy product importer and continue to use international trade as an effective way to balance its domestic demand and supply of dairy products.

## Graphical Abstract

**Figure:**
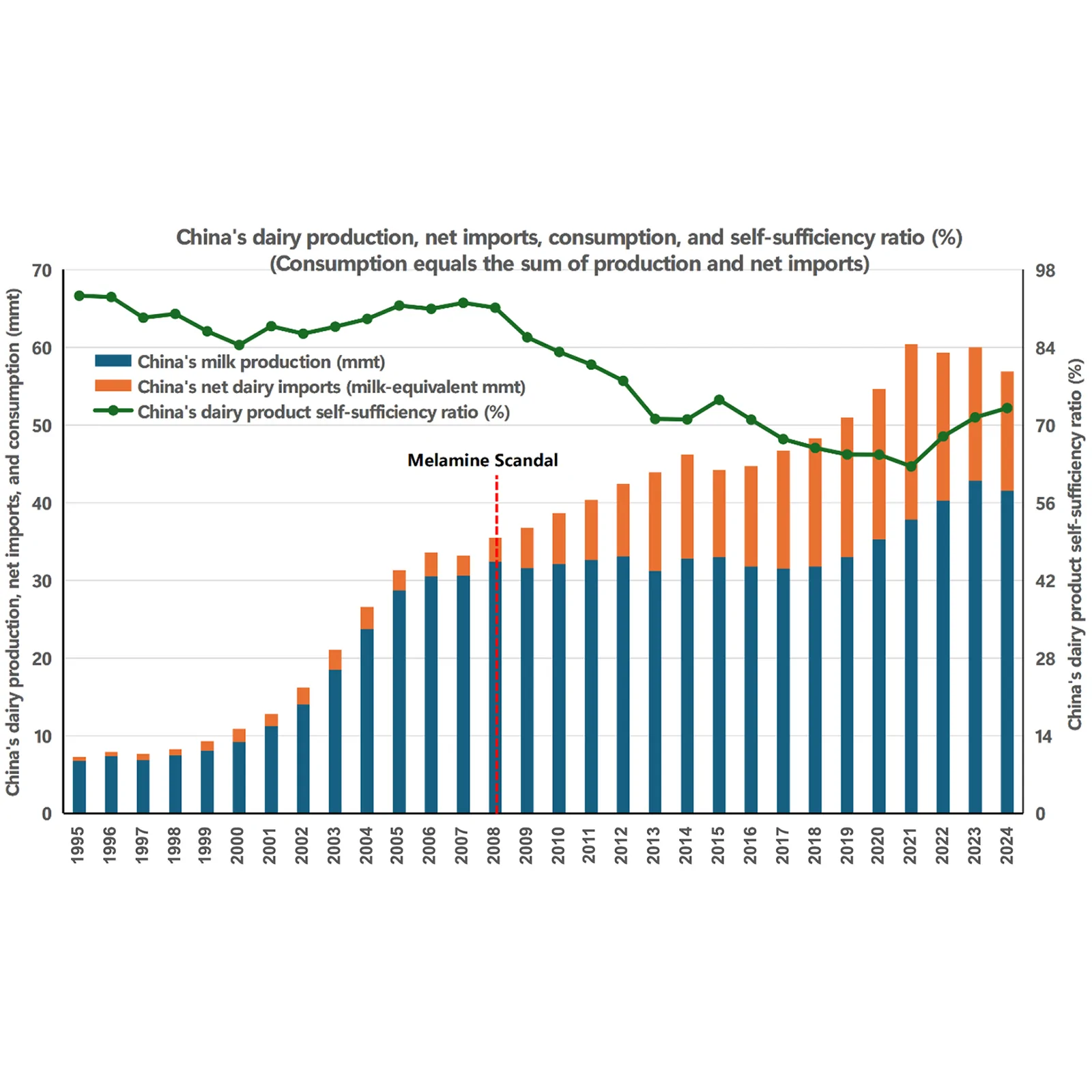

Food safety scandals often involve widespread contamination, fraud, or negligence and cause illness, deaths, massive product recalls, long-term market stagnation, and significant regulatory changes (Soon et al., 2020; Wiśniewska, 2023, De Oleo et al., 2024). Well-known food safety scandals include the Spanish toxic oil syndrome in 1981, where industrial rapeseed oil, denatured with aniline, was illegally refined and sold as olive oil that poisoned more than 20,000 people and resulted in roughly 1,000 deaths (Woolf, 2022; Benito-León et al., 2025); the 2008 melamine scandal in China that sickened more than 300,000 infants and caused 6 deaths (World Health Organization, 2009); the 2013 European horse meat scandal, where beef products were found to contain horse DNA across multiple supermarkets in the United Kingdom and Ireland (Smith and McElwee, 2021; van der Meulen, 2025); and the German *Escherichia coli* outbreak in 2011, where contaminated fenugreek sprouts from Egypt led to a massive outbreak that resulted in approximately 50 deaths (Bielaszewska et al., 2011; Rasko David et al., 2011). In the United States, major food safety scandals include the 1993 Jack in the Box *E. coli* outbreak (Murano et al., 2018), the 1993 Jensen Farms Listeria outbreak (McCollum et al., 2013), and the 2008 to 2009 Peanut Corporation of America *Salmonella* outbreak. Although the amount of literature on different aspects of food safety scandals and outbreaks is robust, this study contributes to the literature by empirically assessing the long-term effects of China's 2008 melamine scandal on its milk production, dairy product consumption and imports, and self-sufficiency ratio using the time-series data from 1995 to 2024.

China's 2008 melamine incident was one of the most severe food safety scandals in China and around the world. As a nitrogen-rich industrial chemical that can cause kidney damage and other health problems, especially for infants, melamine was purposefully added to watered-down milk by some dairy farmers and suppliers to artificially inflate the protein content to pass the quality control checks because standard milk testing at the time measured milk's protein content based on its nitrogen content (Jia et al., 2012; Rovina and Siddiquee, 2015; Li et al., 2019a). Following many reports from Hebei and other provinces, the central Chinese government officials notified the public on September 16, 2008, that infant milk formulas produced by 22 domestic dairy companies had been contaminated with melamine, and it was later revealed that many other domestically produced dairy products, including fluid milk and yogurt, were also contaminated with the industrial chemical (Xiu and Klein, 2010; Huang, 2014). Among the companies that produced contaminated infant milk formula and other dairy products, the primary culprit was the Sanlu Group, one of the largest dairy producers in China (Huang, 2014). Twenty-one other domestic companies, including Mengniu and Yili, were also found to have been producing contaminated milk or infant milk formulas (Xiu and Klein, 2010; Li et al., 2019b). By November 2008, an estimated 300,000 babies had fallen ill, with more than 50,000 hospitalizations and 6 deaths, from consuming the contaminated infant formulas (World Health Organization, 2009).

The melamine scandal caused dramatic drops in consumer trust in domestically produced dairy products, and many consumers stopped purchasing domestic milk and dairy products, especially infant formulas, once the scandal was widely reported across China (Qiao et al., 2012; Huang, 2014; Soon-Sinclair et al., 2024). As a result, China's imports of dairy products, especially infant formulas, milk powder, and whey, started to increase rapidly in 2009. In response to the domestic milk production stagnation, rapidly increased dairy product imports, and financial challenges facing the whole dairy industry, the Chinese government created and implement new food safety regulations and many other measures around 2010 (Qian et al., 2011; Jia et al., 2012; Huang, 2014). Although the government interventions, including new regulations, testing procedures, and financial support for facility upgrades, helped to mitigate drops in herd sizes and farmer participation in the dairy industry in some regions (Jia et al., 2012), China's total domestic milk production did not recover to the 2008 level until 2019. The stagnation in domestic milk production was mainly due to the slow recovery in consumer trust in domestic milk and other dairy products (Yin et al., 2018; Li et al., 2019b, 2021). Even by the mid-2010s, many Chinese consumers, especially parents with infants, still did not trust domestically produced infant milk formulas and other dairy products and were willing to pay more for imported dairy products (Dong and Li, 2016; Yin et al., 2018; Li et al., 2019b, 2021). For example, Dong and Li (2016) compared urban consumers' dairy product purchases before and after the melamine scandal and found that dairy consumption had not recovered within 5 yr following the scandal in some cities. Li et al. (2021) found that consumer trust in government and third parties increased because of their role in food safety regulation and inspections, but they remained distrustful of dairy farmers, processors, and retailers because of concerns about contaminants, false advertising, and selling of expired products.

Although previous studies have documented the melamine scandal's effects on consumer confidence and milk production in China, most of the studies on milk production focused on the short-term effects (Qiao et al., 2010; Qian et al., 2011; Wei and Liu, 2012), and empirical studies are lacking on the scandal's long-term effects on China's dairy production, consumption, imports, and self-sufficiency. This study contributes to the literature by assessing such long-term effects using data from 1995 to 2024. Specifically, this study addresses the following research questions raised by dairy farmers, processors, and policymakers who are interested in China's dairy market and its global impacts. (1) Has China's dairy industry recovered from the melamine scandal? (2) How has the melamine scandal affected China's dairy product imports and self-sufficiency? (3) What lessons and policy implications can we derive from the melamine scandal for China's dairy industry to move toward more sustainable development?

Time-series data were collected from multiple sources. For example, China's milk production data from 1981 to 2024 was from China's Statistical Yearbook and China's Agricultural Yearbook published by the Chinese government annually (State Statistical Bureau of China, 2026), and data on China's imports of major dairy products from 1995 to 2024 was collected from the United Nations' Comtrade Database (https://comtradeplus.un.org/) and the USDA Economic Research Service (USDA-ERS; https://www.ers.usda.gov/data-products/state-agricultural-trade-data). The quantities of processed dairy products such as cheese and milk powder were converted into fluid milk-equivalent quantities using the average conversion ratios from USDA (2025) and previous studies.

Graphical analysis was conducted to examine the changes and trends in China's milk production, dairy product imports, and consumption in milk-equivalent quantity, as well as the dairy product self-sufficiency ratio since 1995. Note that the total consumption in milk-equivalent quantity is calculated from the sum of domestic milk production and net dairy product imports in milk-equivalent quantity (in million metric tonnes, **mmt**).

To examine the impact of the melamine scandal's long-term effects on China's total milk production and total dairy product consumption in milk-equivalent quantities, the following regression models are specified:

[1] *Q_t_* = *a* + *b*_1_*T_t_* + *b*_2_*D_mt_* + *b*_3_*D_pt_* + *b*_4_*T_t_ × D_mt_* + *b*_5_*T_t_ × D_pt_* + *e_t_*,

[2] *C_t_* = *k* + *c*_1_*T_t_* + *c*_2_*D_nt_* + *c*_3_*D_kt_* + *c*_4_*T_t_ × D_nt_* + *c*_5_*T_t_ × D_kt_* + *n_t_*,

where *Q_t_* and *C_t_* are the total milk production and dairy product consumption in milk-equivalent quantity in the *t*th year; *T_t_* is a time variable; *D_mt_*, *D_pt_*, *D_nt_*, and *D_kt_* are dummy variables for 2009 to 2018, 2019 to 2024, 2009 to 2021, and 2022 to 2024, respectively; *a*, *b_1_* to *b_5_*, *k*, and *c_1_* to *c_5_* are the coefficients to be estimated, and *e_t_* and *n_t_* are the error terms. The dummy variables were defined to capture the effects of potential shocks and structural changes, such as the 2008 melamine scandal, observed from the time-series data of milk production and dairy product consumption.

The time variable is included in the production model as a proxy variable to represent technical progress and other factors that have contributed to the steady growth in China's milk production since 1995 (Hongli et al., 2020). Although many other factors, including public and private investment, have likely contributed to the steady growth of China's dairy industry since the 1980s (Qian et al., 2011; Hu et al., 2021), most of them are closely correlated with the time variable. Also, data for measuring such factors is very limited. The time variable in the consumption model is a proxy variable to represent the steady growth in average income, increasing consumer preferences for dairy products, improving availability of dairy products in both urban and rural areas, and so on. Per capita income and other variables were not included in the consumption model because of their close correlations with the time variable. The 4 dummy variables were defined based on the preliminary findings from our graphical analysis of the time-series data of milk production and dairy product consumption. For example, China's total milk production increased steadily from 1981 to around 1995, grew at a significantly higher rate from 1995 to approximately 2008, fluctuated along a flatline from 2009 to 2018, and finally started to grow in 2019 and kept a steady and significant growth rate until it dropped slightly in 2024. Based on the review of the development patterns and trends of China's dairy industry since the early 1980s, it seems reasonable to hypothesize that the 2008 melamine scandal was the major factor for the stagnation of China's milk production from 2009 to 2018. However, the exclusion of many concurrent factors should be considered as a limitation of this study and the empirical findings from the production and consumption models should be interpreted with caution.

Note that the interaction terms between the time variable and dummy variables are included to assess the melamine scandal's effects on milk production and dairy product consumption as well as whether China's milk production and dairy product consumption, respectively, have recovered to their growth rates before the scandal. For example, if the estimated value of *b*_4_ is negative and significantly different from 0, it shows the negative effect of the melamine scandal on China's annual growth rate of milk production, estimated for 1995 to 2008, during the period from 2009 to 2018. Also, if the estimated value of *b*_5_ is not significantly different from 0, it suggests that the annual growth rate of China's milk production after 2019 was not significantly different from the annual growth rate estimated for 1995 to 2008.

Figure 1 shows China's milk production from 1981 to 2024 and China's dairy product imports and total consumption in milk-equivalent quantities from 1995 to 2024. Starting at a very low level in 1981, China's milk production increased steadily over the next 15 years, then experienced remarkable growth at an increasing rate from 1995 to the melamine scandal in 2008, fluctuated around a flatline during the next decade, and finally started to grow at a significant rate in 2019, reaching a record high level in 2023 before it dropped slightly in 2024. The growth in China's total dairy product consumption, in milk-equivalent quantity, was consistent with the growth in its milk production because of limited international trade until the melamine scandal in 2008, but then had a totally different growth pattern after 2008. While domestic milk production fluctuated around a flat line from 2009 to 2018, the total dairy product consumption continued to grow steadily until 2022, when the total consumption started to decline. The gaps between total domestic consumption and production were met through dramatically increased dairy product imports from 2009 to 2021. As a result, China has gradually emerged as a large importer of many dairy products since the melamine scandal in 2008.

**Figure 1:**
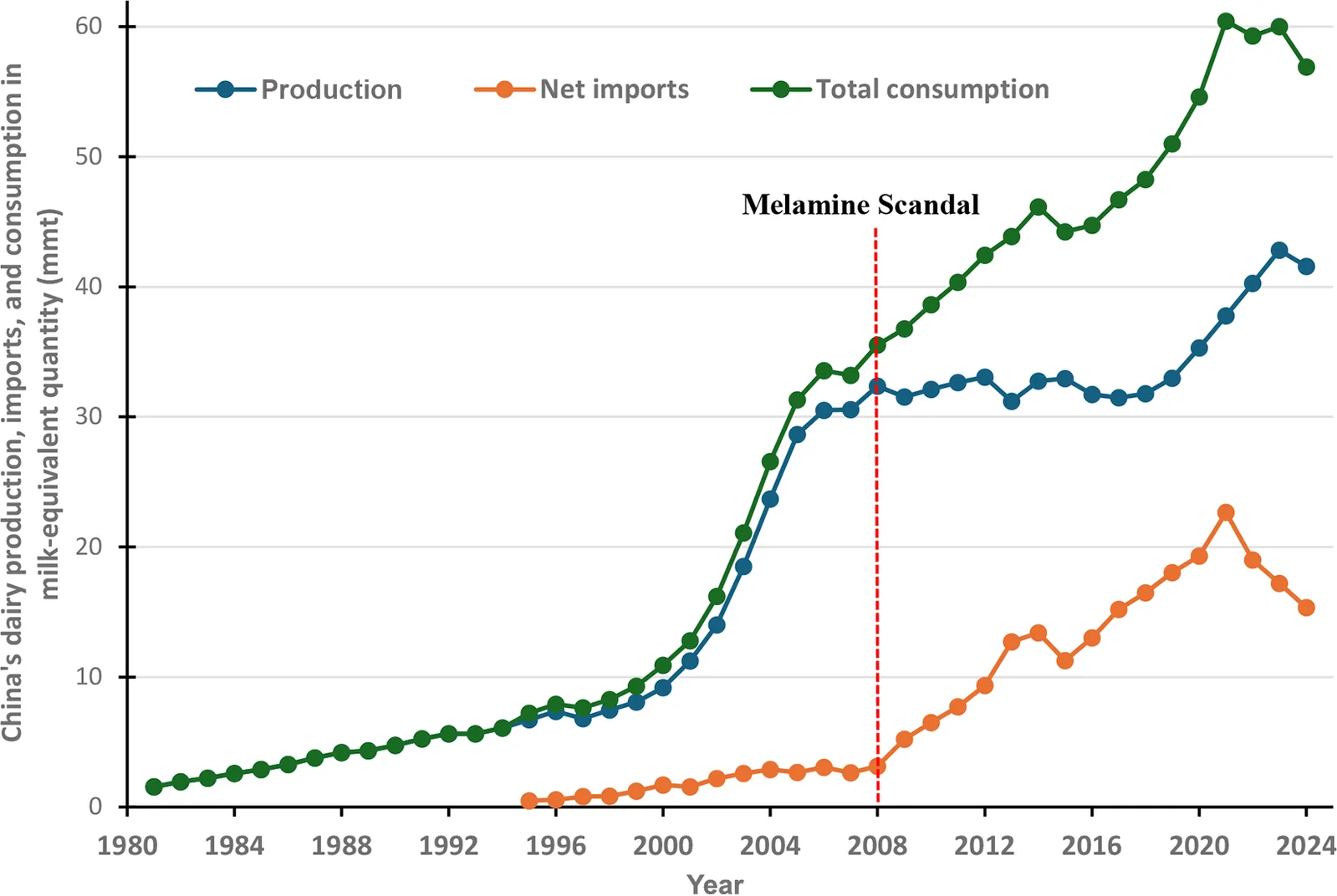
China's dairy production, net imports, and consumption in milk-equivalent quantity (mmt).

Data on China's imports of major dairy products from 1995 to 2024, presented in Figure 2, suggests 3 major findings. First, China's imports of major dairy products increased steadily from 1995 to 2004, then fluctuated around 0.4 mmt in milk-equivalent quantity from 2005 to 2008, increased rapidly and steadily from 2009 to 2021 and reached its record high level in 2021, and then dropped significantly from 2022 to 2024. The decreases from 2022 to 2024 were likely due to a slowdown in China's economic growth and declining birthrates and significant growth of domestic production of milk and many other dairy products (Chu, 2024a; Howard, 2025). Second, whey, skim, and whole milk powder together have dominated China's dairy product imports, making China a large and competitive market for such products. Third, China's cheese import has increased at a steady rate since the late 2000s.

**Figure 2:**
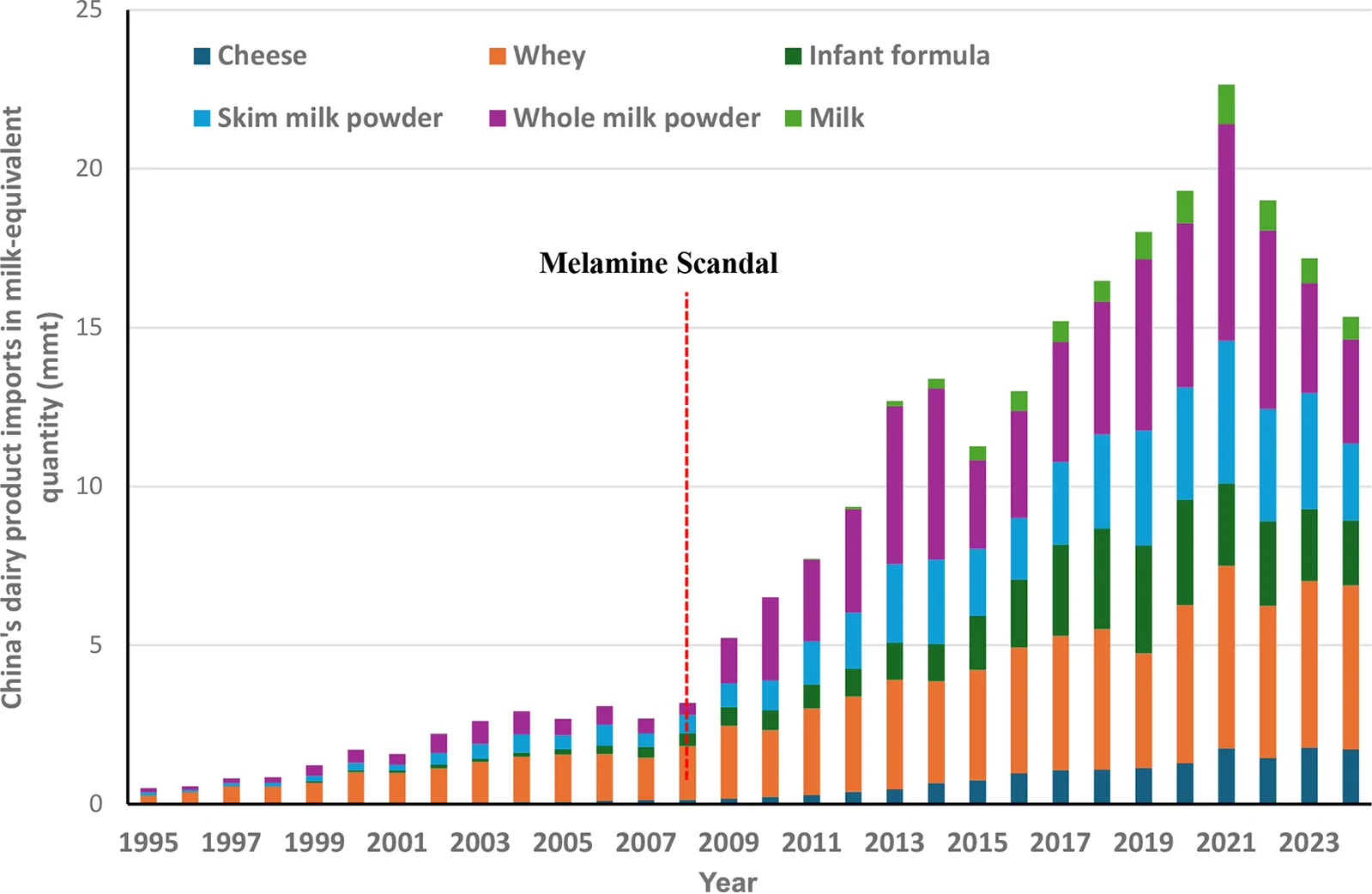
China's imports of major dairy products in milk-equivalent quantity (mmt).

China's total dairy production, import and consumption data, presented in Figure 1, was used to calculate its dairy product self-sufficiency ratio and proportion of imported products in its total consumption. Although China's declining food self-sufficiency ratio had emerged as a major concern for the Chinese government and many researchers, this study provides additional evidence that China's dairy food self-sufficiency ratio dropped significantly from 91.12% in 1995 to a record low of 62.52% in 2021 and then increased to 73.04% in 2024. China's overall food self-sufficiency ratio fell from 94% in 2000 to roughly 66% in 2020, and it is predicted to drop to around 60% by 2030 (Chu, 2024b; Donnellon-May, 2025). Despite its high domestic production, China has emerged as the world's largest importer of many food products (Zhu et al., 2025). For example, approximately 85% to 90% of China's total consumption of soybeans has been imported from the global market in recent years (Jiang et al., 2025). China's massive soybean imports have made it the world's largest soybean importer, accounting for approximately 60% of the global trade in this commodity.

The growing concern about China's food self-sufficiency is largely because of its population size and the increasing geopolitical conflicts and risks (Erokhin et al., 2022; Liang et al., 2023; Chang et al., 2025; Du et al., 2025). The US–China trade war and retaliations, the Russia–Ukraine war, the Iran war, and political tensions between China and Japan highlighted food and energy risks to China and the global supply chains with increasing fears of worsening food and energy insecurity (Wang et al., 2023; Song et al., 2025; Wu and Zhu, 2025).

The estimated regression results for the production model, reported in Table 1, suggest 3 major findings. First, the estimated model fits the data very well and explains 95.7% of the variation in the dependent variable (total milk production) over the period from 1995 to 2024. This goodness of fit is also indicated by the high significant level of the *F*-statistic. Second, China's total milk production increased at an average of 2.31 mmt per year from 1995 to 2008, but the annual growth rate dropped to −0.0359 mmt per year over the next 10 years (2.3100 − 2.3459 = −0.0359) and this is likely a good estimate of the 2008 melamine scandal's huge impact on China's milk production for 10 yr. Third, the estimated coefficient of *T_t_* × *D_pt_* was negative but was not significant at the 0.1 significance level, suggesting that the average annual growth rate rebounded to 1.9409 mmt per year during 2019 to 2024 (2.3100 − 0.3691 = 1.9409), but this result should be interpreted with caution because of the insignificance of the estimated coefficient at the 0.10 significance level. The null hypothesis that the annual growth rate of China's milk production during 2019 to 2024 was the same as the annual growth rate estimated for 1995 to 2008 was not rejected at the 0.01 significance level, suggesting that China's milk production growth rate recovered to the prescandal level during 2019 to 2024.

**Table 1:** Estimated regression models of China's milk production and dairy consumption in milk-equivalent quantity (mmt)

Variable	Definition	Milk production	Significance level	Dairy consumption	Significance level
*T_t_*	= 1 for 1995, = 2 for 1996, ..., = 25 for 2024	2.3100	***	2.2936	***
*D_mt_*	= 1 for 2009–2018 and = 0 otherwise	33.4044	***		
*D_pt_*	= 1 for 2019–2024 and = 0 otherwise	−14.3506	***		
*T_t_* × *D_mt_*	Interaction term of *T_t_* and *D_mt_*	−2.3459			
*T_t_* × *D_pt_*	Interaction term of *T_t_* and *D_pt_*	−0.3691			
*D_nt_*	= 1 for 2009–2021 and = 0 otherwise			2.5594	
*D_kt_*	= 1 for 2022–2024 and = 0 otherwise			88.3614	
*T_t_* × *D_nt_*	Interaction term of *T_t_* and *D_nt_*			−0.3337	
*T_t_* × *D_kt_*	Interaction term of *T_t_* and *D_kt_*			−3.4050	
Constant		−0.5753		1.7362	
R^2^		0.9646		0.9682	
Adjusted R^2^		0.9572		0.9616	
*F*-statistic		130.85	***	146.10	***
N	Number of observations	30		30	

*** *P* < 0.01.

The estimated regression results of the consumption model, reported in Table 1, suggest 3 major findings. First, the adjusted R^2^ and F-statistic indicate that the estimated model fits the data very well and explains 96.16% of the variation in the dependent variable over the study period. Second, the estimated average annual increase in China's total dairy product consumption in milk-equivalent quantity over the period from 1995 to 2008 was 2.29 mmt, which is very close to the average annual increase of 2.31 mmt in total production estimated from the production model. The 2 vales were very close due to China's limited dairy product imports in comparison with its total production and consumption during the period. Third, the 2008 melamine scandal reduced the average annual growth in China's dairy product consumption by 0.3337 mmt over the period from 2009 to 2021, but the estimated effect was not statistically significant at the 0.1 significance level. This finding suggests that the melamine scandal negatively affected the growth in China's dairy product consumption, but the effect was very limited and not significant. Third, the estimated coefficient of *T_t_* × *D_kt_* (−3.4050) suggests that China's total dairy product consumption dropped significantly from 2022 to 2024, but the estimate was not significant at the 0.10 significance level due to the short period of only 3 yr. Potential reasons for the declines in China's total dairy product consumption and imports since 2022 include the slowdown in its income and economic growth in recent years, continuous population decline that started in 2022, significant drops in new births since 2018, and increased availability of many substitutes for dairy products. Future research might investigate whether the changes from 2022 to 2024 were merely short-term changes or represent a long-term trend when more data becomes available.

This study suggests 4 major conclusions. First, while China's total milk production increased by about 2.31 mmt per year from 1995 to 2008, the average annual growth was almost 0 during the next 10 years. Although the 2008 melamine scandal was very likely the key factor for the 10-yr stagnation in China's milk production, it means that it took 10 yr for China's dairy industry to recover from the 2008 melamine scandal. Second, China's dairy product consumption has continued to increase at a steady rate since the 1990s, the melamine scandal's effect on consumption was relatively limited, and the gaps between domestic demand and supply from 2009 to 2021 were met through rapidly increased dairy product imports, from 2.96 mmt in 2009 to 15.57 mmt in 2018, reaching a record high of 22.65 mmt in 2021. Third, the rapid increase in China's imports reduced its dairy product self-sufficiency ratio from 91.12% in 2008 to 65.85% in 2018 and the ratio reached a record low of 62.52% in 2021. Fourth, major factors for the declines in China's dairy product imports and total consumption since 2022 likely include the slowdown in its income and economic growth, continuous population decline starting in 2022, significant drops in new births since 2018, and increased availability of substitute products such as soy milk, but the period is too short to confirm or quantify these factors' effects.

This study suggests 3 major policy implications. First, the effects of the 2008 melamine scandal on Chinese consumer confidence on domestic dairy products and its dairy industry were huge and long lasting, and China must continue to take serious measures to improve its food safety and avoid future scandals. Although the 2008 melamine scandal triggered sweeping policy changes in China, including the 2009 Food Safety Law, which established stricter production, testing, and recall standards, alongside severe penalties—up to the death penalty—for adulteration (Qian et al., 2011; Jia et al., 2012; Lam et al., 2013), China still faces many challenges in improving its food safety in general and dairy food safety in particular. As shown in this study, the effects of government actions on consumer confidence in domestic dairy products and the recovery of the dairy industry were limited for about a decade until the late 2010s. China has a significant and long-term journey to improve its food safety and secure its food supply chain and faces major challenges, including a fragmented industry, weak enforcement of food safety law and regulations at local levels, and persistent safety breaches, requiring improved oversight from farm to table (Wang et al., 2021; Yang et al., 2022; Soon-Sinclair et al., 2024).

Second, China could continue to use international trade as an effective way to balance its domestic demand and supply for dairy products, especially for high-value products like cheese, milk powder, and whey. While fostering self-sufficiency, China has heavily imported milk powder, whey, and specialized dairy products, primarily from New Zealand, the European Union, and the United States, to meet the rising domestic demand, especially when domestic supply faces economic constraints or capacity limits (Wang and Zou, 2020). Third, as a large dairy product importer with more than 1.4 billion consumers, China's growing dairy product markets and changing trade behavior have significant effects and implications for the global dairy product markets and require more attention and studies, especially empirical studies that address market and policy questions.
